# Supplementary material for: Nucleation phenomena and extreme vulnerability of spatial k-core systems
Source: Nat Commun. 2024 Jul 12;15:5850. doi: 10.1038/s41467-024-50273-5 (PMC11239893; doi:10.1038/s41467-024-50273-5)
Supplement: Supplementary file 1 — Supplementary Information [file 41467_2024_50273_MOESM1_ESM.pdf]

SUPPLEMENTARY INFORMATION: Nucleation phenomena and extreme vulnerability of spatial k-core systems

Leyang Xue,[1,2] Shengling Gao,[2,3] Lazaros K. Gallos,[4,*] Orr Levy,[5,6] Bnaya Gross,[2] Zengru Di,[1,†] and Shlomo Havlin[2,‡]

[1]*International Academic Center of Complex Systems, Beijing Normal University, Zhuhai, 519087, China*
[2]*Department of Physics, Bar-Ilan University, Ramat-Gan, 52900, Israel*
[3]*School of Mathematical Sciences, Beihang University, Beijing, 100191, China.*
[4]*DIMACS, Rutgers University, Piscataway, New Jersey 08854, USA*
[5]*Department of Immunobiology, Yale University School of Medicine, New Haven, CT, USA*
[6]*Howard Hughes Medical Institute, Chevy Chase, MD, USA*

The square lattice as a model of k-core percolation

We can demonstrate the basic principles of the k-core percolation process, especially those related to nucleation and hole propagation, by using a simple model of a two-dimensional square lattice, as mentioned in the Model section. Since the initial degree of all nodes is $k = 4$, the system is at a critical state for a 4-core percolation process. Indeed, when we remove one lattice node anywhere in the system (Fig. S1, then each of its four neighbor nodes will lose one link and they will all have a degree $k = 3$. These neighbors form a layer which is removed in the next iteration, so that now the nodes in the next layer will all have also degree less than 4. This process will continue with the propagation of this damage front which will eventually unravel the entire system.

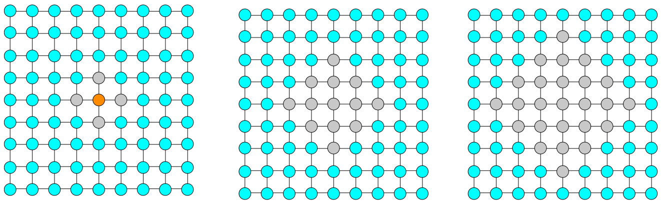

FIG. S1. **The 4-core percolation process in a simple 2D square lattice.** The removal of one node anywhere in the lattice creates a layer of nodes with degree less than $k = 4$, which in turn leads to the removal of successive layers. This hole expands until it consumes the entire system.

The calculation of the largest cluster as a function of the number of iterations is straightforward, since we only have to calculate the number of nodes for each layer that is removed. The number of removed nodes at each time step, S_t, is growing linearly and is equal to $S_t = 4t$ for an infinite lattice. For a finite lattice of linear size, $2L + 1$, this number is equal to:

$$S_t = \begin{cases} 4t & t \leq L \\ 4(2L+1-t) & L < t \leq 2L \end{cases} . \quad (1)$$

Based on this result, we can calculate the total number of failed nodes, $P^f_\infty(t)$, which yields:

$$P^f_\infty(t) = \begin{cases} 2t(t+1)+1 & t \leq L \\ -2t^2 + 2t(4L+1) - 4L^2 + 1 & L < t \leq 2L \end{cases} . \quad (2)$$

We can find the number of nodes in the largest remaining cluster by simply subtracting the number of failed nodes, $P^f_\infty(t)$, from the total number of nodes $N = (2L + 1)^2$, which leads to the following expressions:

$$P_\infty(t) = \begin{cases} (2L+1)^2 - 2t(t+1) - 1 & t \leq L \\ 2t^2 - 2t(4L+1) + 8L^2 + 4L & L < t \leq 2L \end{cases} . \quad (3)$$

This process can be seen as a demonstration of the nucleation and cascading behavior, described in detail in the main text for the ζ-model, and can help visualize how minimal intervention can affect the structure at a global scale. In the case of 4-core percolation, the initial fraction of nodes in the giant component is 1. If we remove a single node anywhere in the lattice the fraction of nodes remains 1 (in an infinite system) but then after each k=4-core pruning step the cascading process removes another internal layer leading to a growing hole which at the equilibrium will lead to the absence of a giant component. In other words, the removal of just a single node leads to a discontinuous transition via the emergence of one giant hole.

The ζ-model and finite size effects

The construction of the spatially embedded networks using the ζ-model is using a 2D square lattice as a substrate. We start by fixing the system size $N = L \times L$, the average degree $\langle k \rangle$, and the value of ζ. The average degree and the system size determine the total number of links in the system as $N\langle k \rangle/2$. For each link, the first step is to generate a link length by selecting random numbers from the exponential distribution for the Euclidean length l, $p(l) \sim e^{-l/\zeta}$, where ζ defines the characteristic length of the links in the network. In the second step, we randomly select one node and determine a candidate node set according to the value of the random link

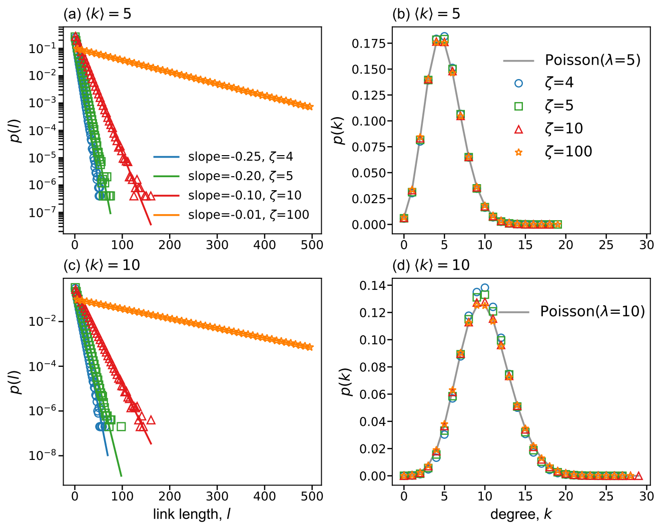

FIG. S2. **The probability densities of the link length, $p(l)$, and of the degree, $p(k)$, are not influenced by the network construction method.** The link length distribution, $p(l)$, is shown for different values of ζ, for (a) $\langle k\rangle = 5$ and (c) $\langle k\rangle = 10$. Similarly, the resulting degree distribution, $p(k)$, remains close to the Poisson distribution with parameter λ represented with a solid line, for (b) $\langle k\rangle = 5$ and (d) $\langle k\rangle = 10$.

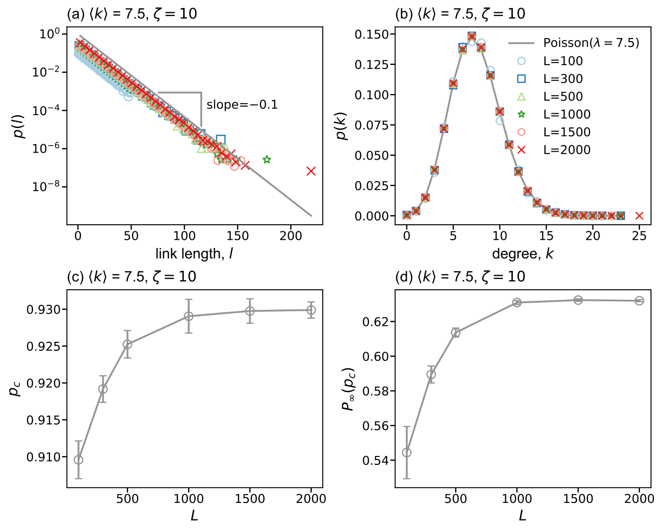

FIG. S3. **The effect of system size on the model and on percolation properties.** (a) The exponential distribution of link lengths is not influenced by the system size. (b) The degree distribution remains invariant on system size changes and λ remains the same. (c) The value of the critical point, p_c, converges at $L = 1000$. (d) The fraction of nodes in the giant connected component at the critical point, $P_\infty(p_c)$, also converges at $L = 1000$. All results are obtained for a network with $\langle k\rangle$=7.5 and ζ=10. Each point in (c) and (d) represents the average over 200 realizations for $L < 1000$, 100 realizations for $L = 1000$, and 25 realizations for $L > 1000$, with the error bars indicating the standard deviation.

length. The final step is to randomly select one node from this candidate set and establish the link between the two nodes. We repeat the above process until all links are assigned. The resulting degree distribution satisfies a Poisson distribution, $P(k) = \lambda^k e^{-\lambda}/k!$ with a mean degree $\lambda = \langle k\rangle$, since the links are assigned randomly. Following the network construction method described in the main text, we tested whether the resulting empirical distributions follow the initially assigned probability densities. In Fig. S2 we present different $P(k)$ and $p(l)$ distributions for different combinations of ζ and $\langle k\rangle$, where it is seen that both the degree distribution and the link distribution are not influenced by the network construction model.

We also tested the percolation results for convergence as a function of the network linear size, L. In Fig. S3 we present these results for linear sizes ranging from $L = 100$ to $L = 2000$. The link length distribution already converges for $L = 300$, while the degree distribution seems invariant in this range. The value of the percolation critical point, p_c, however, changes slightly from 0.91 at $L = 250$ to 0.93 at $L = 1000$, and remains constant for larger system sizes. Similarly, the fraction of nodes at the largest component converges at $L = 1000$. Based on these findings, all our results in the main text use a network with $L = 1000$.

The effect of the characteristic link length on k-core percolation

For k-core percolation with $k < 3$, the critical point decreases with increasing ζ, and a continuous phase transition is observed at all characteristic lengths, as shown in Figs. S4(a) and S5(a)(b). While long-range links can facilitate the spread of failure across a broader network region, the system for $k \leq 2$ remains resilient with no extensive cascading failures (by definition, the k-core condition does not impose additional restrictions for node removal in 1-core and 2-core percolation, compared to the percolation condition of belonging to the largest cluster) [1]. For k-core percolation with $k \geq 3$, e.g. $k = 3, 4, 6$ in Fig. S4, long range interactions lead to the phenomena described in the main text.

Spatio-temporal propagation of a hole

To illustrate the spatio-temporal propagation of cascading failures, we analyzed the radius r_h of holes that emerged during the dynamic process. Figures S6(a)-(c) show an example of the hole radius evolution, $r_h(t)$. In Fig. S6(d), we plot r_h against t for various ζ values, where we can see that r_h transitions from a low value to a larger

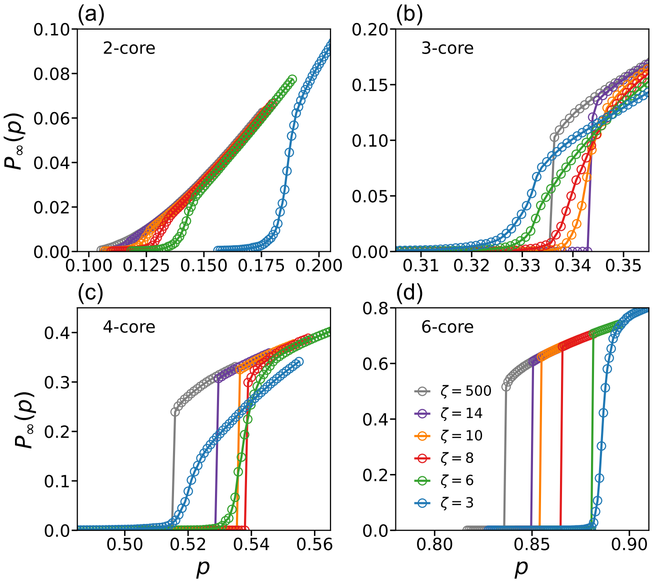

FIG. S4. **The effect of characteristic link length on k-core percolation.** (a)-(d) The giant component size P_∞ as a function of p for various k-core percolation (i.e. k=2, 3, 4,and 6). For $k \geq 3$, the system shows a change from a continuous to a discontinuous phase transition as ζ increases. However, for $k < 3$, the giant component exhibits a continuous phase transition across all ranges of ζ values. Network configurations and number of realizations are the same as in Fig. 1 of the main text.

constant value via almost linear growth. In all cases, two distinct states are observed, which correspond to critical branching and to the nucleation process. At low r_h values, the system undergoes a critical branching process where only small holes exist that grow slowly, and there are no holes of critical finite size. Once the hole size reaches the critical finite size, r_h exhibits a linear growth associated with a nucleation process. This nucleation process continues as r_h increases until r_h reaches a constant value when the holes reach the system boundary. Interestingly, as ζ increases, it takes fewer time steps to form holes with a critical finite size, the duration of the nucleation process is shorter, and the hole grows faster than in smaller ζ. The speed of the hole growth is plotted in Fig. S6(e), where we can see that this speed increases linearly with the characteristic link length ζ. As explained in the main text, the nucleation process becomes weaker as ζ moves away from ζ_c.

Differences in the dynamic evolution for small and large characteristic link lengths

To provide further insight into the distinction between nucleation and critical branching processes, we follow the evolution in two extreme cases, $\zeta = 10$ and $\zeta = 100$, in addition to the cases of $\zeta = 7$ and $\zeta = 500$ shown in

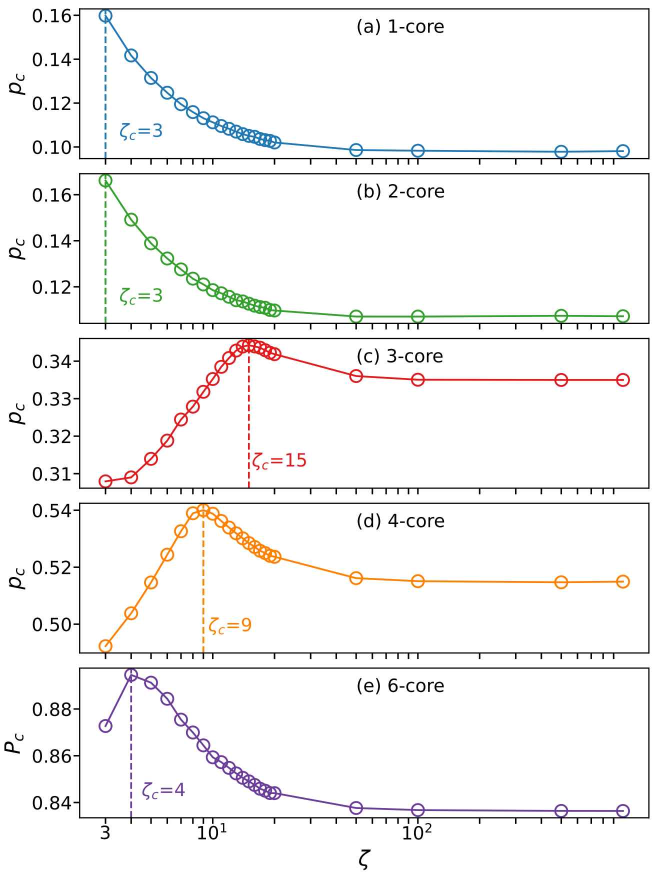

FIG. S5. **The critical point changes with characteristic link length ζ for different k-core percolation.** For 1-core and 2-core percolation, the system exhibits a continuous phase transition across all values of ζ. For $k \geq 3$, the point which separates second-order and first-order phase transitions corresponds to the maximum p_c, identified as the critical characteristic length of link ζ_c. Increasing the k-core value results in a smaller ζ_c value, because larger k-core percolation leads to stronger localized failures, requiring shorter links to trigger catastrophic failures. The results are averaged over 50 realizations.

Fig. 3. When ζ is close to ζ_c, the radius of the hole that can trigger the failure propagation is close to the minimal radius value. Hence, holes that emerge during the dynamic process quickly reach the critical size and induce the nucleation process in subsequent time steps. As observed in Figs. S7(a)(c)(e) for $\zeta = 10$, one can see that $P_\infty(t)$ undergoes a shorter branching process and then decreases parabolically, while $P^f_\infty(t)$ increases parabolically until the system collapses. In contrast, when ζ is of the order of the system length L, as shown in Figs. S7(b)(d)(f), we observe a prolonged plateau for $P_\infty(t)$, followed by a sharp decrease and an abrupt growth for $P^f_\infty(t)$, associated with an avalanche process. As p gradually moves away from p_c, both the nucleation and the critical branching processes exhibit a behavior similar to that in p_c, but with shorter dynamic

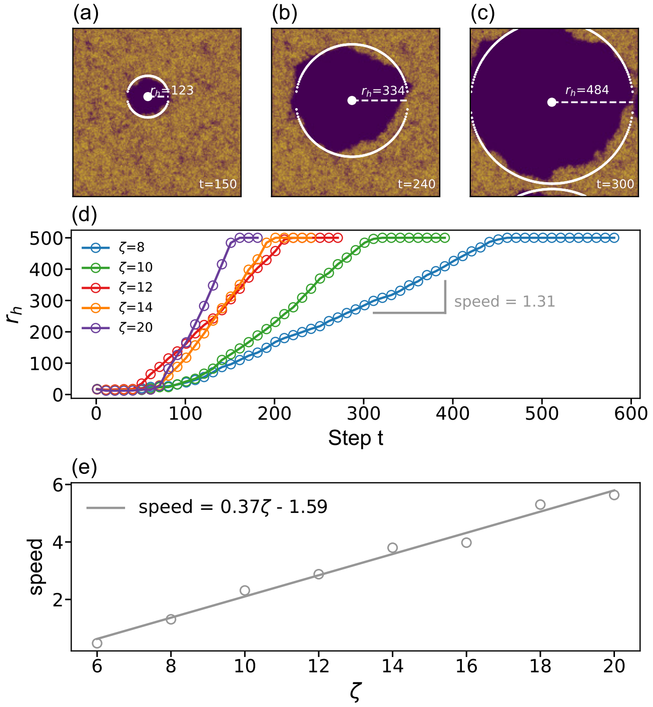

FIG. S6. **Temporal spatial spreading process of holes**. (a)-(c) Schematic of holes with radius r_h during the dynamics process at $\zeta = 10$, displayed for three snapshots of time t = 150, 240, and 300. We track here the maximum hole numerically and measure its radius. (d) Evolution of the maximum hole size, r_h, with time t for 5-core percolation. As ζ approaches ζ_c, the nucleation process lasts longer, and the critical branching process becomes shorter. (e) The speed of the hole is calculated via the slope of the lines in panel (d).

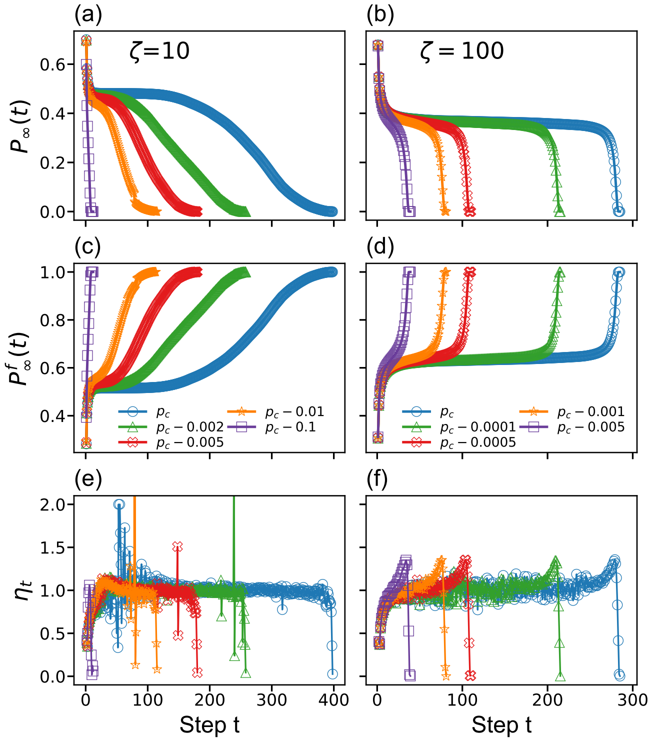

FIG. S7. **Evolution of cascading failure in 5-core percolation**. The left column, (a), (c), (e) corresponds to $\zeta = 10$, and the right column, (b), (d), (f), to $\zeta = 100$. (a)(b) Giant component size $P_\infty(t)$ as a function of the k-pruning time step. (c)(d) The size of the largest failure cluster at time step t. (e)(f) The branching factor as a function of time step t, is defined as the ratio of the failure size between two consecutive steps at t and $t-1$.

processes.

The dynamic process and mechanisms driving the phase transition for various ζ

In k-core percolation, the nature of the phase transition depends on the characteristic length, ζ, and on the existence of spontaneous fluctuations which form due to the node removal process. Values of ζ below ζ_c lead to a continuous phase transition. At $\zeta \sim \zeta_c$ the transition changes to first order, and for even higher values of ζ of the order of the system size, the transition changes to mixed-order. By examining snapshots at different time steps, here we give a clear insight into the underlying mechanisms behind the dynamic processes which lead to the corresponding phase transitions, as shown in the Fig. S8.

For small ζ values, failures are limited to local propagation. As shown in Fig. S8(a1)-(a3), the damaged regions of the network gradually propagate outward from the initial points of failure, leading to a structure exhibiting typical fractal features. In line with this, in Fig. S8(a), the size of the giant component exhibits an almost parabolic decrease, while the largest failure cluster shows parabolic growth in earlier time steps. This is followed by a prolonged plateau, indicating the removal of a small number of nodes (microscopic sizes) at each step, as evidenced in Fig. S8(a) and (a4)-(a9).

For intermediate ζ values, local failures can propagate further. Local fluctuations give rise to a hole, i.e. a large local concentration of removed nodes. The formation of such a hole is followed by an expansion of the hole diameter, as it consumes nodes around its perimeter which continues to grow until the entire system collapses, see Fig. S8(b2)-(b9). Figure S8(b) clearly illustrates a rapid decrease in the size of the giant component as the hole expands outwards, coupled with a corresponding growth in the size of the largest failure cluster, a behavior consistent with a nucleation process.

For larger ζ values, of the order of the system size, failures can transfer anywhere in the system. The network structure remains homogeneous throughout the removal process, without any significant holes, which indicates a bifurcation process. The network continuously dilutes until it eventually undergoes an avalanche process which

leads to a sudden collapse, as evidenced in Fig. S8(c1)-(c9). As a result, the size of the giant component and the largest failure cluster display an extended plateau phase, which ultimately leads to rapid descent and growth, respectively.

Localized attacks in the metastable phase are independent of the system size

A key finding in this work is that a localized attack of a small critical size can destroy the system, independently of the system size. We demonstrate this point in Fig. S9, where we plot the critical radius length, i.e. the minimum hole size r_h^c that can destroy the system, as a function of the average degree $\langle k \rangle$ and of the characteristic length, ζ, for different system lengths (see also Figs. 4(c) and (d) in the main text). These plots are the equivalent of a phase diagram, where the structural system parameters (here $\langle k \rangle$ and ζ) determine the state (phase) of the system. In general, we can distinguish three different regimes: (a) *Unstable* regime. At the bottom of the diagram, $r_h^c \sim 0$ which means that the system is already at a collapse state. (b) *Stable* regime. A stable regime appears at the top area of the diagram, which corresponds to dense stable networks with high $\langle k \rangle$ values. The structure in the original system is now stronger and nodes have a higher number of connections, which means that they are less vulnerable to the loss of a neighbor. As a result, the critical hole size is of the order of the system size $r_h^c \sim L$, i.e. the system cannot be destroyed by a localized attack, except for the trivial case where this attack reaches the entire system. (c) *Metastable* regime. In this area, the critical hole size, r_h^c, has a finite value smaller than the system linear scale. The key observation here is that r_h^c remains constant in this regime as we increase the system size in the figure. We observe a small finite-size effect, where the extent of the regime becomes smaller with size, but it is clear that there is always an area where the value of r_h^c remains unchanged. The same behavior is also observed for any k-core value. These observations demonstrate the extreme vulnerability of the system to localized attacks in this metastable regime, since a very large system can fail due to removing a very small fraction of its nodes.

* Corresponding author. Email: lgallos@gmail.com
† Corresponding author. Email: zdi@bnu.edu.cn
‡ Corresponding author. Email: havlins@gmail.com

[1] Shengling Gao, Leyang Xue, Bnaya Gross, Zhikun She, Daqing Li, Shlomo Havlin, 2024. Possible origin for the similar phase transitions in k-core and interdependent networks. *New Journal of Physics*, 26(1):013006. IOP Publishing.

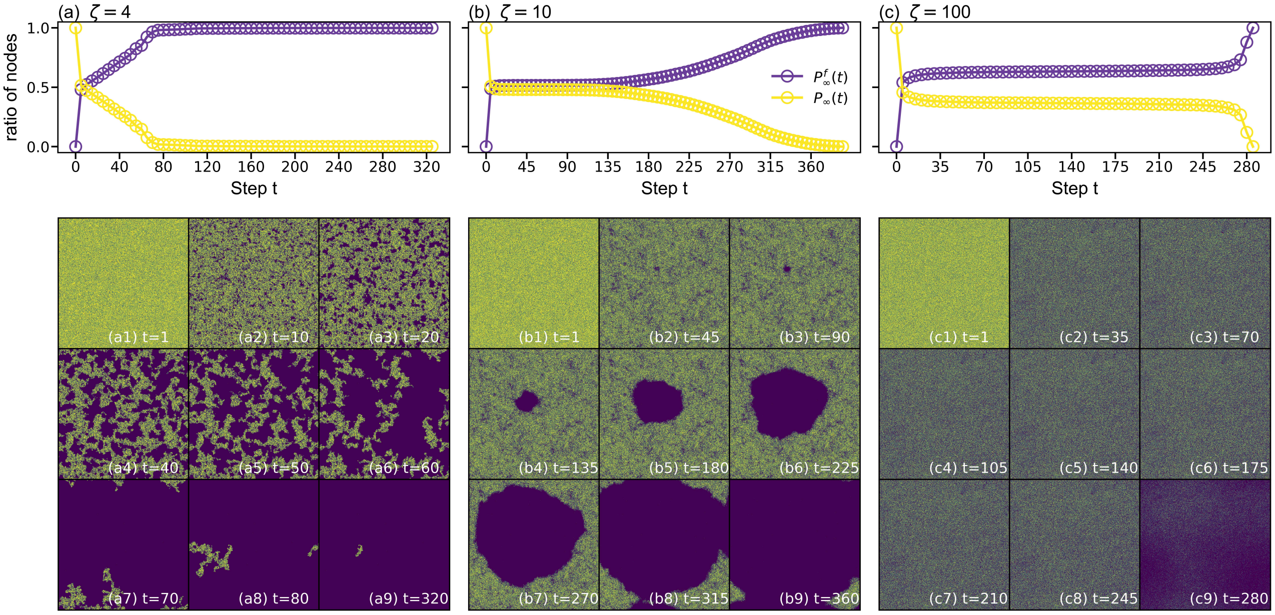

FIG. S8. **Evolution of cascading failures for three different phase transitions in 5-core percolation: continuous, first-order and mixed-order transitions.** The ratio of nodes in the giant component ($P_\infty(t)$) and the largest failure cluster ($P^f_\infty(t)$) are shown for (a) $\zeta = 4$, (b) $\zeta = 10$, and (c) $\zeta = 100$ as a function of time step t. Snapshots of the largest failure cluster at various time steps are also shown for (a1)-(a9) $\zeta = 4$, (b1)-(b9) $\zeta = 10$ and (c1)-(c9) $\zeta = 100$. Purple color represents the failed nodes that constitute the largest failure cluster, while yellow color denotes the nodes that do not belong to the largest failure cluster. Here, we display the dynamic process which leads to complete collapse in a simulation performed at criticality.

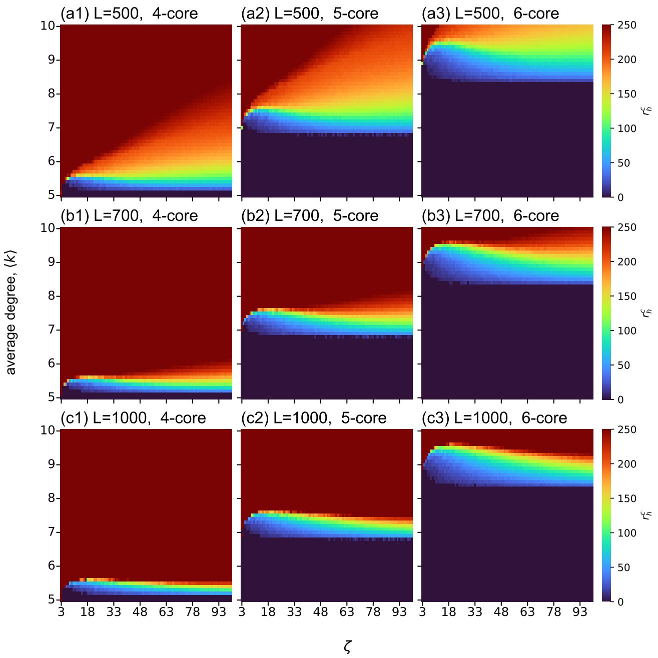

FIG. S9. **Finite-size effect in the critical hole size, r_h^c.** We plot the critical hole size, r_h^c, as a function of ζ and the average degree $\langle k \rangle$ for different k-core percolation systems. To highlight regions that are not influenced by system size changes, we fix the range of r_h^c from 0 to 250. The left column, (a1), (b1), (c1) corresponds to 4-core percolation, the middle column, (a2), (b2), (c2) corresponds to 5-core percolation, and the right column, (a3), (b3), (c3), corresponds to 6-core percolation. (a1)-(a3) $L = 500$. (b1)-(b3) $L = 700$. (c1)-(c3) $L = 1000$.
